# COVID-19: A master stroke of Nature

**DOI:** 10.3934/publichealth.2020033

**Published:** 2020-06-22

**Authors:** Sushant K Singh

**Affiliations:** Artificial Intelligence and Analytics, Healthcare and Life Science, Virtusa Corporation, New York, NY, USA

**Keywords:** COVID-19, coronavirus, pandemic, public health, Nature, sustainability

## Abstract

This article presents the status of countries affected by COVID-19 (as of mid-May 2020) and their preparedness to combat the after-effects of the pandemic. The report also provides an analysis of how human behavior may have triggered such a global pandemic and why humans need to consider living sustainably to make our future world livable for all. COVID-19 originated in the city of Wuhan, China in December 2019. As of mid-May, it has spread to 213 countries and territories worldwide. The World Health Organization has declared COVID-19 a global pandemic, with a death toll of over 300,000 to date. The U.S. is currently the most impacted country. Collaborative efforts of scientists and politicians across the world will be needed to better plan and utilize global health resources to combat this global pandemic. Machine learning-based prediction models could also help by identifying potential COVID-19-prone areas and individuals. The cause of the emergence of COVID-19 is still a matter of research; however, one consistent theme is humanity's unsustainable behavior. By sustainably interacting with nature, humans may have avoided this pandemic. If unsustainable human practices are not controlled through education, awareness, behavioral change, as well as sustainable policy creation and enforcement, there could be several such pandemics in our future.

## 1. Introduction

The concept of sustainable development is to act responsibly so that earth will continue to be livable for future generations. However, the definition of sustainable development itself has become questionable because the goals are not to make the Earth sustainable, instead of adding more millennium sustainable goals [1]. Considerable evidences suggest that humans have not learned from past experiences, including from failures of sustainable development and more than a thousand years of global pandemics [1]–[3]. Through the 2019 coronavirus disease (COVID-19), fast becoming one of the deadliest global pandemics, nature has again offered humans an opportunity to learn the value of sustainable living practice.

## 2. A snapshot of historical global pandemics

On 11 March 2020, the World Health Organization (WHO) declared COVID-19 a global pandemic [4]. This designation was made after 118,000 reported cases of COVID-19 in 114 countries worldwide [4]. The world has encountered multiple deadly pandemics, from the Antonine Plague (also known as the Plague of Galen), in AD 165 that killed five million people and annihilated the Roman army though to the COVID-19 pandemic (Table 1) [5]. History records at least 20 global pandemics with an estimated total death toll of over 400 million people (Table 1) [5].

**Table 1.** A summary of the global pandemics.

Sl. No.	Name	Start period	End period	Type/Pre-human host	Death toll
1	Antonine Plague	165	180	Believed to be either smallpox or measles	5,000,000
2	Plague of Justinian	541	542	Yersinia pestis bacteria/Rats, fleas	50,000,000
3	Japanese smallpox epidemic	735	737	Variola major virus	1,000,000
4	Black Death	1347	1351	Yersinia pestis bacteria/Rats, fleas	200,000,000
5	New World Smallpox Outbreak	1520	Continued	Variola major virus	56,000,000
6	Italian plague	1629	1631	Yersinia pestis bacteria/Rats, fleas	1,000,000
7	Great Plague of London	1665	1665	Yersinia pestis bacteria/Rats, fleas	100,000
8	Yellow Fever	1800	1800	Virus/Mosquitoes	150,000
9	Cholera Pandemics 1–6	1817	1923	V. cholera bacteria	1,000,000
10	Third Plague	1885	1885	Yersinia pestis bacteria/Rats, fleas	12,000,000
11	Russian Flu	1889	1890	Believed to be H2N2 (avian origin)	1,000,000
12	Spanish Flu	1918	1919	H1N1 virus/Pigs	50,000,000
13	Asian Flu	1957	1958	H2N2 virus	1,100,000
14	Hong Kong Flu	1968	1970	H3N2 virus	1,000,000
15	HIV/AIDS	1981	Continued	Virus/Chimpanzees	35,000,000
16	SARS	2002	2003	Coronavirus/Bats, Civets	770
17	Swine Flu	2009	2010	H1N1 virus/Pigs	200,000
18	Ebola	2014	2016	Ebolavirus/Wild animals	11,000
19	MERS	2015	Continued	Coronavirus/Bats, camels	850
20	COVID-19	2019	Continued	Coronavirus-Unknown (possibly pangolins)	296,067

The Black Death, which occurred between 1347 and 1351 has been the deadliest pandemic with a death toll of 200 million people, followed by smallpox in the New World, the Plague of Justinian, the Spanish Flu, and HIV/AIDS (Table 1) [5]. Today, the newly emergent COVID-19 is a severe global health threat, with new cases of COVID-19 and deaths still exponentially increasing.

## 3. Current global status of the COVID-19 pandemic

COVID-19 first originated in December 2019 in the city of Wuhan, China, and was initially identified in a series of pneumonia patients [6],[7]. Later, it was discovered that the disease was caused by a newly identified β-coronavirus, designated severe acute respiratory syndrome coronavirus 2 (SARS-Co-2) [6]. At first, the disease was named 2019 novel coronavirus (2019-nCoV) but was later designated COVID-19 by the WHO [6],[7]. Bats (*Chiroptera*) have been reported to be a natural host of SARS-CoV-2 and the virus might have been spread through transitional hosts, such as domestic pets or wild animals used as food sources [6]. COVID-19 spreads through human contacts [6].

**Figure 1.**
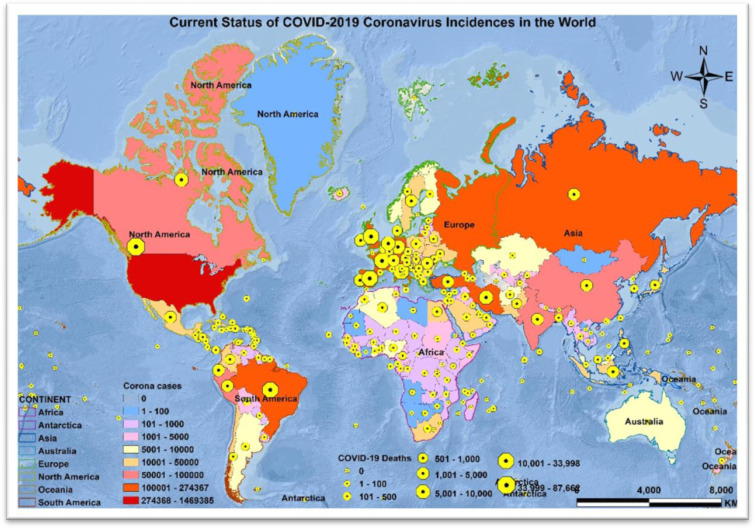
Current status of COVID-19 coronavirus incidences and deaths in the world.

As of 13 May 2020, at 5:00 PM EST, 4,398,078 people have tested positive for COVID-19. Of these, 2,462,922 are active cases and 1,935,156 are closed cases, of which 296,067 had died and 1,639,089 had recovered from the disease (Figure 1) [8],[9].

The first case of COVID-19 was internationally reported on 10 January 2020, in the city of Wuhan, China. Within three months, it had spread to 213 countries and territories (Figure 1). The Diamond Princess cruise ship harbored in Yokohama, Japan, and Holland America's M.S. Zaandam cruise ship were two international cruise ships on which 712 and 9 people tested COVID-19 positive, and 11 and 2 patients died, respectively [8],[9].

### 3.1. The ten countries most affected by COVID-19

Out of 213 affected countries and territories, the United States of America (U.S.), Spain, Russia, the United Kingdom (U.K.), Italy, Brazil, France, Germany, Turkey, and Iran are, as of mid-May 2020, the most severely impacted countries, with collectively 72% of the total cases, 80% of the total deaths, 65% of the new deaths, and 56% of the new cases of COVID-19. These countries contain 68% of total recovered patients; however, 74% of the active cases and 84% of critical patients are also in these countries [8],[9]. Many of the 213 affected countries are underdeveloped and lack essential resources, which is a cause for concern. The level of preparedness in each country is also an important consideration, and can be approximated.

**Figure 2.**
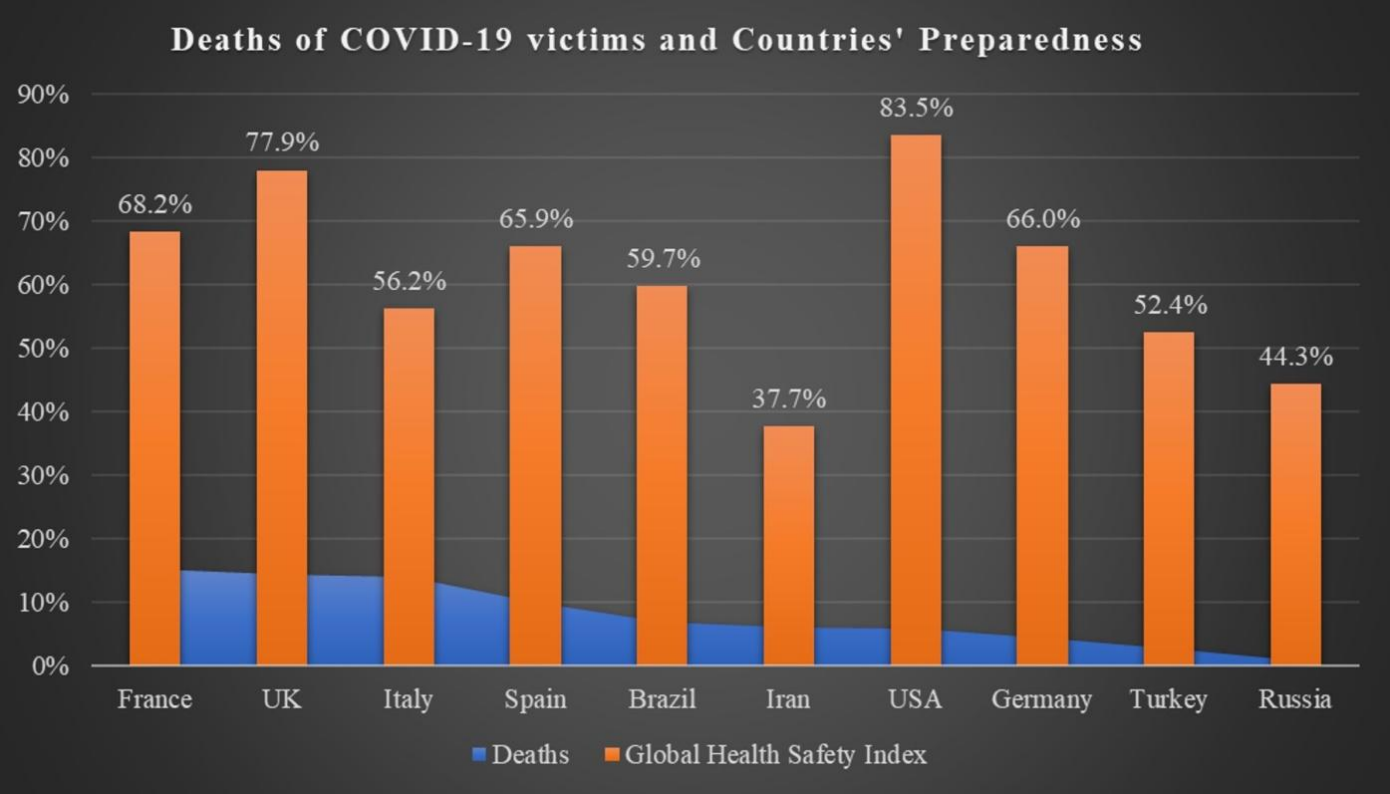
Death of COVID-19 victims and countries' preparedness. Note: Most prepared: Score 66.7 to ≥ 100; more prepared: Score 33.4 to ≥ 66.6; least prepared: Score 0 to ≥ 33.3.

Using the Global Health Security Index (GHSI), as a metric of preparedness, the country with the highest percentage of total deaths out of total cases among the top ten affected countries was reported to be France (deaths = 15%; GHSI = 68.2), followed by the U.K. (deaths = 13%, GHSI = 77.9), Italy (deaths = 13%; GHSI = 56.2), Spain (deaths = 10%; GHSI = 65.90), Brazil (deaths = 7%; GHSI = 59.7%), Iran (deaths = 6%; GHSI = 37.7%), the U.S. (deaths = 4%; GHSI = 83.5%), Germany (deaths = 2%; GHSI = 66.0), Turkey (deaths = 3%; GHSI = 52.4%), and Russia (deaths = 1%; GHSI = 44.3%) (Figure 2) [10]. Although several of these countries have a lower GHSI, in general these countries have a high gross domestic product (GDP). The U.S., U.K., and France were the most prepared to combat the COVID-19 pandemic, based on GHSI.

Comparing the gross domestic product (GDP), as an indicator of preparedness through resource availability, the U.S. (GDP = $21.44 trillion; rank = 1^st^) seems to be the most capable of combating the existing challenges as well as the after-effects of the COVID-19 pandemic [11]. Germany (GDP = $3.8 trillion; rank = 4^th^), the U.K. (GDP = $2.83 trillion; rank = 6^th^), France (GDP = $2.71 trillion; rank = 7^th^), and Italy (GDP = $2.07 trillion, rank = 8^th^) may be able to cope with the existing challenges but may struggle post-COVID-19 [11]. Brazil (GDP = $1.8 trillion; rank = 9^th^), Russia (GDP = $1.6 trillion; rank = 11^th^), and Spain (GDP = $1.3 trillion; rank = 13^th^) may also manage in the current emergency situation, however, considering their preparedness and their GDP they may have significant post-COVID-19 impacts [11]. The lower relative economic prosperity of Turkey (GDP = $743 billion; rank = 19) and Iran (GDP = $463.08 billion; rank = 27^th^) could significantly impact their coping capacity in the existing situation as well as after the pandemic settles [11].

On the other hand, countries with some of the highest percentage of the total reported deaths out of total reported cases, for example, Nicaragua (deaths = 32%; GHSI = 43.1), Mauritania (deaths = 22%; GHSI = 27.5), Sint Maarten (deaths = 20%, GHSI = NA), Yemen (deaths = 17%; GHSI = 18.5), Antigua and Barbuda (deaths = 12%; GHSI = 29), Bahamas (deaths= 12%; GHSI = 30.6), Chad (deaths = 11%; GHSI = 28.8), Belize (deaths = 11%; GHSI = 31.8), and Zimbabwe (deaths = 11%; GHSI = 38.2) have a lower GHSI that indicates that they are poorly prepared for the COVID-19 pandemic [11]. The adage that the poor suffer the most is likely to prove true in respect to these countries. Most of these countries also have very low nominal GDP. Consequently, they are extremely vulnerable to the current situation of the COVID-19 pandemic, and they may struggle to cope with its economic after-effects [11]. How these nations react in response to the pandemic will also be critical. For example, Italy, with over 31 thousand deaths, initiated a nationwide lockdown after 600 people died. Following Italy's course, France, Spain, New Zealand, Belgium, the U.K., South Africa, Colombia, Bolivia, Jordan, and Tunisia also implemented nationwide quarantine policies. India, where the COVID-19 pandemic is at its early stages, announced its nationwide lockdown for three weeks on 22 March 2020, with 1,024 cases and 27 deaths (now total cases = 78,055 and deaths = 2,551). On the contrary, even with the highest number of COVID-19 cases and more than 20,000 deaths as of mid-May 2020, the U.S. did not declare nationwide lockdown. Therefore, the availability of resources may not ensure the safety of human lives in these pandemic areas rather proactiveness could control further spread of this infectious disease [12].

## 4. A masterstroke of nature

Human and natural systems interrelate in a variety of ways and *Homo sapiens* would not survive a major disruption of natural cycles of oxygen, carbon, nitrogen, phosphorus, sulfur, and water [13]. Scientific and social communities have also suggested that a “reconnect to nature” is warranted [14]. Research suggests that a disconnection with nature could lead to grave consequences for humanity, for example, loss of the ability to respond effectively to adverse natural conditions [15]. It has been reported that over 75% of all new transmittable viruses come from wildlife [16].

The origin of COVID-19 is assumed to be through natural selection where either the virus could have evolved in a non-human host and then been transmitted to humans, or a non-pathogenic form of it could have been transmitted from a non-human host and then evolved to its pathogenic form in humans [17]. Since similar coronaviruses are found in wild mammals including pangolins (*Pholidota*), and bats, they are proposed to be natural reservoirs of COVID-19. It is highly likely that several animal species may be natural hosts for these viruses, which may be transmitted to humans in the future [17]. Despite several viral outbreaks, bats have been eaten in Africa, Latin America, Caribbean, and East Asian countries for decades [18]. In China, at least 54 different species of reptiles and mammals, including cats, dogs, rats, and bats are legally allowed for consumption. The consumption of bats has continued in Indonesia since the outbreak of the COVID-19 pandemic [19],[20].

There is an increasing trend towards adoption of the “Paleo Diet” (Paleolithic Diet, also known as the Caveman Diet, the Stone Age Diet or the Hunter-Gatherer Diet), primarily inspired by the assumption that meat, a low-carbohydrate food source, can help in weight loss, improved health, and longevity [21]. In contrast to the “Paleo Diet”, humans two million years ago ate primarily leaves, fruits, wood, and bark [22]. Another study reported that over 105,000 years ago, the Mozambique people may have survived on the cereal grass sorghum [23]. Reports also suggest that in various locations, ancient people were dependent on plant foods by choice, as opposed to by necessity [24],[25].

The argument here is not to mandate that people go vegan or vegetarian, but instead that humans should interact more harmoniously with nature. With globalization and the worldwide interaction of cultures, it is not feasible to scan each traveler at every airport or shipping port to check for the presence of a virus. Instead, it would be prudent to avoid all consumption of wild animals, animals known to harbor potential human pathogens, and animals not part of the food habits of our distant ancestors. Although some tribal populations are forced by necessity to eat bushmeat, the consequences of wider consumption can have fatal worldwide consequences.

The COVID-19 pandemic could be considered as a masterstroke of nature, in its devastating effect across even economically strong countries with leading healthcare systems. Infection of visible figures such as political leaders (including the U.K. Prime Minister Boris Johnson) and celebrities (including Hollywood star Tom Hanks) have highlighted that the virus has spared neither rich nor poor [26]. COVID-19 has forced nationwide lockdown in many countries, with the suspension of industry and transport that is reported to have triggered improvements in the ozone layer in the Southern Hemisphere [27], and led to various accounts of improving biodiversity across the world. These indications suggest the benefits of a harmonious interaction with nature; COVID-19 provides a warning case-in-point of the dangers of acting otherwise.

## 5. Prevention through prediction

In five months since the first reports of COVID-19, a vast amount of data has been generated that could help in developing prediction models using various mathematical, statistical or machine learning techniques. Prediction models could help in identifying potential areas or individuals susceptible to outbreaks of COVID-19. Machine learning techniques have been used in numerous contexts, such as predicting environmental contaminants, natural hazards, an individual's awareness of environmental contamination, and a person's likelihood of adopting mitigation technologies [28]–[32]. Likewise, several prediction models have been developed on COVID-19 data. For example, digital signal processing is used for genome analyses and to classify the coronavirus by applying decision tree algorithms [33]. In another study, a deep learning model was developed for precisely distinguishing between COVID-19 infection and community-acquired pneumonia using patients' tomography images [34]. Similarly, logistic regression models were developed using the epidemiological and clinical features of COVID-19 patients [35]. A short review of studies of various prediction models used for COVID-19 can be found here [36]. However, a comprehensive prediction model is still lacking and requires inputs from various scientific as well as social and political experts.

The vulnerability of an area or an individual is multidimensional and it is a combined effect of the exposure to COVID-19; sensitivity to COVID-19 infection due to socioeconomic, demographic, psychological, health, and geographic factors; and the adaptive or coping capacities to combat the challenge [37]. As a global problem, COVID-19 needs a global solution: it would be more accurate and effective if worldwide consortiums of experts were formed to address the challenges [38]. In the last five months a lot has been learned about the infectious behavior of the causative virus SARS-CoV-2, communities' response to COVID-19 spread, the preparedness of local and global health institutions to combat the pandemic, and the actions and reactions of local and global political entities [39]. Therefore, the characteristics of the sensitivity, exposure, and coping capacity to COVID-19 have the potential to be defined. For example, social, behavioral, and mental health problems due to COVID-19 have emerged as major challenges [40]. Most of the current prediction models are focused on one aspect of COVID-19; they are based on small datasets, or the datasets have a few predictors. A global consortium could help develop more inclusive as well as realistic regional or global prediction models of COVID-19. This global collaboration could also help avoid any data redundancy or gaps and allow for development of a real-time decision support system for COVID-19 incidences. The decision support system could be developed with open access tools and could be freely available to the scientific community as well as to the general public [41].

## 6. Conclusion

The COVID-19 global pandemic has been devastating, with 300,000 people killed in 5 months, leaving the countries with the largest economies on the back foot. The world continues to struggle to adapt to the challenge and each individual is learning how to survive in the global crisis. Sustainable living must be a key outcome of this global health problem, and future generations can be protected as follows:

- Globalization should be used to promote sustainability as opposed to trading.
- Resources should be utilized for sustainable education, awareness, and behavioral change as opposed to heavy investment in atomic and other military powers.
- Healthcare systems should be strengthened, especially in the low GDP countries.
- All animals that are known carriers for potential pathogenic human viruses should be banned globally for hunting and food.
- Social bonding and behavioral strengths should be enhanced by following sustainable social and behavioral habits.
- Cutting-edge computational methods should be used to develop accurate and robust prediction models.
- A global consortium of scientists and policy experts should be established to research and respond to the COVID-19 pandemic.
- Open access technologies should be used for the creation of a global pandemic dashboard, to be made available to the world free of cost.
- Most importantly, people should act responsibly and make informed decisions.
